# The relative contributions of frontal and parietal cortex for generalized quantifier comprehension

**DOI:** 10.3389/fnhum.2014.00610

**Published:** 2014-08-07

**Authors:** Christopher A. Olm, Corey T. McMillan, Nicola Spotorno, Robin Clark, Murray Grossman

**Affiliations:** ^1^Penn Frontotemporal Degeneration Center, Department of Neurology, Perelman School of Medicine, University of PennsylvaniaPhiladelphia, PA, USA; ^2^Department of Linguistics, University of PennsylvaniaPhiladelphia, PA, USA

**Keywords:** quantifiers, number knowledge, language, semantic memory, fMRI

## Abstract

Quantifiers, like “some” or “few,” are frequent in daily language. Linguists posit at least three distinct classes of quantifiers: cardinal quantifiers that rely on numerosity, majority quantifiers that additionally depend on executive resources, and logical quantifiers that rely on perceptual attention. We used BOLD fMRI to investigate the roles of frontal and parietal regions in quantifier comprehension. Participants performed a sentence-picture verification task to determine whether a sentence containing a quantifier accurately describes a picture. A whole-brain analysis identified a network involved in quantifier comprehension: This implicated bilateral inferior parietal, superior parietal and dorsolateral prefrontal cortices, and right inferior frontal cortex. We then performed region-of-interest analyses to assess the relative contribution of each region for each quantifier class. Inferior parietal cortex was equally activated across all quantifier classes, consistent with prior studies implicating the region for quantifier comprehension due in part to its role in the representation of number knowledge. Right superior parietal cortex was up-regulated in comparison to frontal regions for cardinal and logical quantifiers, but parietal and frontal regions were equally activated for majority quantifiers and each frontal region is most highly activated for majority quantifiers. This finding is consistent with the hypothesis that majority quantifiers rely on numerosity mechanisms in parietal cortex and executive mechanisms in frontal cortex. Also, right inferior frontal cortex was up-regulated for logical compared to cardinal quantifiers, which may be related to selection demands associated with logical quantifier comprehension. We conclude that distinct components of a large-scale fronto-parietal network contribute to specific aspects of quantifier comprehension, and that this biologically defined network is consistent with cognitive theories of quantifier meaning.

## INTRODUCTION

Quantifiers are extraordinarily common in language. Phrases such as “at least three,” “less than half,” and “some” are very frequent. In formal semantic terms, these phrases serve the function of asserting a property about a set and mapping it to a truth-value. For example, in the phrase “at least three blue birds,” the property is the number of blue birds and the truth-value here is whether this quantity of blue birds falls above the limit “three.” While we know a great deal about the formal semantic properties of generalized quantifiers (Barwise and Cooper, 1981; van Benthem, 1986), we understand little about the neural basis for quantifier comprehension. In this study, we examine the neuroanatomic basis for quantifier comprehension in healthy adults with fMRI.

There are several classes of quantifiers (Clark and Grossman, 2007). Cardinal quantifiers such as “at least three” have an explicit number component. Comprehension of a cardinal quantifier thus depends on the ability to consider the number specified in the quantifying phrase, enumerating the relevant target states, and then comparing that quantity to the quantifier. “Majority” quantifiers like “most” or “less than half” involve a number component and additionally depend on computational resources such as strategic processing and working memory before the truth statement can be evaluated. To evaluate a majority quantifier like “less than half of the birds are blue,” the total number of relevant states under consideration (for example, eight birds) must first be evaluated, then the number of states required to meet the criterion in the quantified phrase must be computed (half of eight is four) and maintained in an active state in working memory, then the target states must be enumerated (three blue birds), and finally the quantified target states are compared with the relevant states maintained in working memory (three is less than four) to evaluate the truth-value of the quantifier. By comparison, Aristotelean, or “logical”, quantifiers like “some” and “not all” require evaluation of whether one or more objects are contained within, or absent from, a set. For example, “some” involves detecting a group of targets; finding a single exception is necessary for “not all” to be true.

Past work in patients has shown an association between parietal disease and quantifiers, implicating number knowledge in the comprehension of quantifiers. Studies in non-aphasic patients show that disease in the inferior parietal lobe interferes with number knowledge (Cipolotti et al., 1991; Takayama et al., 1994; Martory et al., 2003; Halpern et al., 2004; Baldo and Dronkers, 2007; Koss et al., 2010). Consistent with the idea that number knowledge contributes to the comprehension of quantifiers, disease in this parietal region in non-aphasic patients with corticobasal syndrome (CBS) also interferes with the comprehension of cardinal quantifiers, logical quantifiers, and majority quantifiers that depend in part on number knowledge (McMillan et al., 2005, 2006; Troiani et al., 2009a).

Previous patient work also has related prefrontal regions to the comprehension of majority quantifiers, implicating executive resources in the comprehension of quantifiers. Prefrontal regions have been associated with executive deficits on measures of working memory (Kimberg and Farah, 1993; Bechara et al., 1998; Thompson-Schill et al., 1999; Possin et al., 2013) and strategic processing (Sirigu et al., 1995; Amunts et al., 2004; Possin et al., 2009). Additionally, comprehension of majority quantifiers and logical quantifiers is compromised in non-aphasic patients with prefrontal disease due to the behavioral variant of frontotemporal degeneration (bvFTD; McMillan et al., 2005; Troiani et al., 2009a), and presumably the executive deficits in these patients interferes with their comprehension of these quantifiers.

Few fMRI studies have examined the neuroanatomic basis of quantifiers. Previous work has demonstrated activation of inferior parietal lobe for cardinal and majority quantifiers (McMillan et al., 2005; Troiani et al., 2009a; Heim et al., 2012). We reasoned that this was due in part to the dependence of these quantifiers on number knowledge, a domain also associated with the inferior parietal lobe. There has been substantial fMRI work demonstrating the contribution of the parietal lobe to number knowledge and simple arithmetical calculation. When participants were asked to consider either number symbols (represented by Arabic numerals or number words) or non-symbolic numerical patterns (e.g., 1 cm filled dot arrays), for example, activation was found in bilateral inferior parietal lobe (Dehaene et al., 2003; Shuman and Kanwisher, 2004; Ansari et al., 2006; Castelli et al., 2006; Piazza et al., 2006, 2007; Wei et al., 2012). Further, the role of executive resources in processing majority quantifiers appeared to prompt recruitment of prefrontal regions (McMillan et al., 2005; Troiani et al., 2009a). We and others have also reported logical quantifiers, or alternatively defined sets of quantifying conditions encompassing logical quantifiers, recruiting prefrontal regions (Troiani et al., 2009a; Wei et al., 2012) as well as inferior parietal regions (McMillan et al., 2005; Wei et al., 2012).

Together, these prior findings suggest that there is a large-scale fronto-parietal network that supports the comprehension of quantifiers, and that the relative contribution of parietal and prefrontal cortex activation is modulated by quantifier class. Specifically, parietal cortex appears to support the numerosity component of quantifier comprehension and prefrontal cortex appears to support executive and attentional demands. Prior experiments have focused on whole-brain voxel-wise analyses of patients and have not directly evaluated the relative contribution of each neuroanatomic region to the comprehension of each class of quantifier. In this study, we present the same visual stimuli from a patient study used to probe for cardinal, majority, and logical quantifiers in an fMRI study of healthy young adults, and we explicitly evaluate the relative contributions of parietal and prefrontal cortex activation in region-of-interest (ROI) analyses for each quantifier class. Moreover, we used very small numerosities to minimize the potential confound that may be associated with activation for number knowledge in the same brain region. We hypothesized that inferior parietal lobe activation would be present for all quantifiers due to the role of this region in supporting number knowledge, a crucial component of quantifier meaning, and that prefrontal activation would be more associated with executive resources that play a role in majority and logical quantifiers.

## MATERIALS AND METHODS

### PARTICIPANTS

We recruited 17 right-handed young adults (10 females) from the University of Pennsylvania community, with a mean age of 23.1 years (SD = 2.98, range 18–28) and a mean education of 15.7 years (SD = 2.11, range 12–20). One additional participant was recruited but excluded from all analyses due to excessive movement. All participants were volunteers recruited in accordance with a protocol approved by the University of Pennsylvania Institutional Review Board including an informed consent procedure, and they were financially compensated for their participation.

### EXPERIMENTAL TASK

**FIGURE 1:**
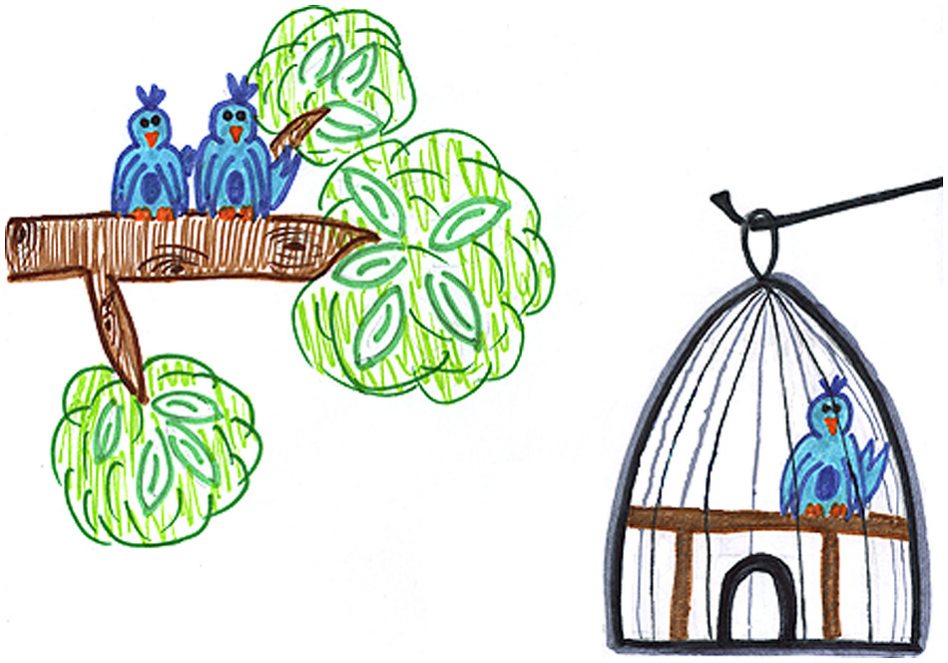
**Sample true-response stimulus material for a logical quantifier (“There are some birds in the tree”), a precise quantifier (“There are two birds in the tree”), cardinal quantifier (“There is at least one bird in the tree”), and a majority quantifier (“More than half of the birds are in the tree”)**.

We generated a total of 120 quantifier stimulus items equally distributed across three conditions: cardinal (e.g., “at least three birds are in the cage”), majority (e.g., “at least half of the birds are in the cage”), and logical (e.g., “Some of the birds are in the cage”). An additional 40 “precise” number stimulus items were generated for a high-level baseline condition, which contained an explicit number in the absence of a quantifier (e.g., “Two birds are in the cage”). These stimuli used small numbers within the range of the approximate number system to minimize any potential confound associated with larger numerosities processed by the precise number system. We also generated 40 filler items that required a true/false response but did not contain a number or a quantifier (e.g., “Birds are in the cage”). Each of the 200 total sentence materials was paired with a colored picture depicting a small number (<5) of familiar objects (e.g., birds) in a natural context (e.g., in a cage; see **Figure 1** for sample stimulus materials). There were a total of 40 unique pictures repeated equally across each quantifier and baseline condition. Half of the sentence and picture pairs were true and half were false.

Stimulus materials were embedded in a list of null event procedures and presented in a randomized order using an event-related design implemented in E-Prime. Participants were first presented with a fixation and sentence for 3000 ms and then a sentence and picture for 3000 ms. The participants viewed sentence materials on a back-projected screen and were instructed to respond using a fiber optic response pad to indicate whether they thought the sentence accurately described the picture. Prior to scanning, the participants were presented with a trial practice session and were given feedback about their performance. We did not provide feedback during the fMRI task to minimize a potential confound of negative or positive reward. Stimulus materials were counterbalanced across five experimental blocks, each containing 40 trials and lasting approximately 7 min. Participants were provided a short, 2 min break between each block.

### IMAGE ACQUISITION

Images were collected using a 3-T Siemens Tim Trio scanner. Each session started with acquisition of a high-resolution T1-weighted MPRAGE structural scan (TR = 1620 ms, TE = 3.09 ms, 192 × 256 matrix). For each experimental block we acquired 144 BOLD volumes using an EPI sequence (TR = 3 s, TE = 30 ms, flip angle = 90^∘^, 64 × 64 matrix, fat saturated), providing 42 axial slices (with no slice gap) consisting of 3.0 mm isotropic voxels per TR.

### IMAGE ANALYSIS

All preprocessing and analysis was performed using SPM8 (http://www.fil.ion.ucl.ac.uk/spm/software/spm8) software. Images were reconstructed from their native DICOM format to NIFTI file format. We then modeled the data for each individual subject. We performed high-pass (>128 s) filtering to remove low-frequency drift. We modeled portions of the time series that correspond to correct responses to stimuli for each subject. All BOLD whole-brain volumes then were realigned to the first volume in the time series (Friston et al., 1995) and then co-registered with the high-resolution structural volume (Ashburner and Friston, 1997). We then warped each individual’s structural volume into MNI152 space using tissue probability maps and then applied these warps to each functional volume for that individual. During spatial normalization, functional data were interpolated to 2 mm isotropic voxels and then smoothed using a 9 mm full-width-half-maximum Gaussian kernel. Lastly, we performed a quality control procedure to assess images with excessive motion or inhomogeneities and excluded these volumes from our analyses. Exclusion criteria were determined by an analysis of more than 160 continuous healthy young adult functional data sets and determining the mean and standard deviations in each of the 6^∘^ of motion and in mean image intensity. We limited volume-to-volume deviations to 3.5 standard deviations from the mean (intensity difference limit = 6.920 (arbitrary units); limits of translations, rotations: *x* = 0.084 mm, 0.00657 radians, *y* = 0.275 mm, 0.00236 radians, *z* = 0.383 mm, 0.00195 radians). In one case, >50% of scans had excessive movement or inhomogeneities, so we excluded this participant from the analysis.

The *t*-test module in SPM was used to compare the contrast between all quantifier conditions (cardinal, majority, and logical) relative to the precise number baseline condition. Because of observed response latency differences for different quantifiers in our data (see below), we removed response time as a covariate. We did not also covary for unequal task accuracy because the hypothesized executive demands associated with different classes of quantifiers were captured more sensitively by response latency. For this contrast we report a height threshold of *p* < 0.05 (uncorrected) with a 20 adjacent voxel extent and only accept clusters exceeding a peak voxel threshold of *p* < 0.001. We used this liberal threshold in an effort to identify all clusters contributing to quantifier comprehension without risking the possibility that marginally sub-threshold clusters would be excluded. To assess the relative contribution of the observed whole-brain contrast clusters we generated the mean beta-value within each independent cluster that we treated as a ROI. We then performed ROI analyses using SPSS 20 (IBM, Chicago) software and used Bonferroni correction for all comparisons.

## RESULTS

### BEHAVIORAL RESULTS

Overall, participants had excellent accuracy, but were less accurate evaluating majority quantifiers (*M* = 85.44%, SEM = 0.02) in comparison to cardinal [*M* = 95.59%, SEM = 0.01; *t*(16) = 4.87; *p*<0.001] and logical quantifiers [*M* = 97.21%, SEM = 0.01; *t*(16) = 6.39; *p* < 0.001]. Accuracy did not differ between cardinal and logical quantifiers [*t*(16) = 1.95, *p* = 0.069].

We observed a similar pattern for response latencies: responses were slower for majority quantifiers (*M* = 1797 ms, SEM = 121) compared to cardinal [*M* = 1261 ms, SEM = 82; *t*(16) = 7.28; *p* < 0.001] and logical quantifiers [*M* = 1293 ms, SEM = 86; *t*(16) = 6.71; *p* < 0.001]. Response latencies did not differ between cardinal and logical quantifiers [*t*(16) = 0.75; *p* = 0.46].

### IMAGING RESULTS

**FIGURE 2:**
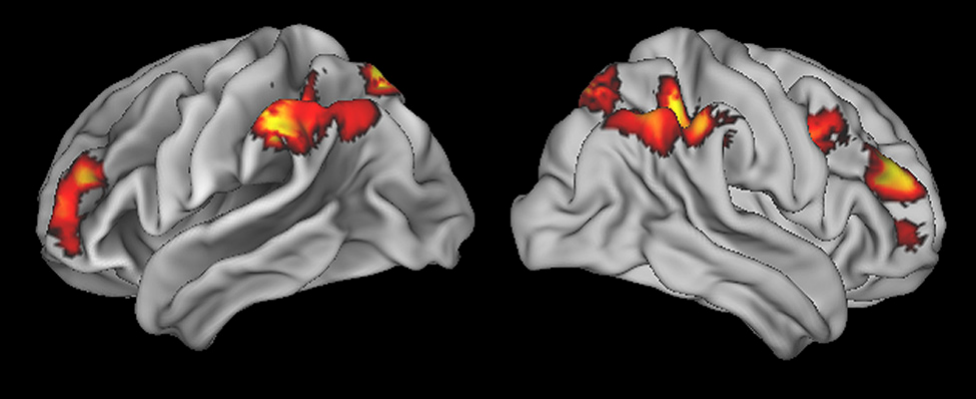
**Regions of activation for all quantifier conditions relative to precise number baseline**.

To identify the fronto-parietal network that supports quantifier comprehension, we first performed a whole brain analysis evaluating activation for all quantifiers (cardinal, majority, and logical) relative to the precise number condition used as a high-level baseline. This contrast revealed activation in seven clusters (see **Table 1** for a summary and **Figure 2** for an illustration of activated clusters). We used the SPM Anatomy toolbox (Eickhoff et al., 2005, 2006, 2007) to localize results. These included four parietal clusters in right inferior parietal, left inferior parietal, right superior parietal, and left superior parietal cortices. Right inferior parietal activation was present in PFm and PGa (Caspers et al., 2008). PF, PFa, PFm, (Caspers et al., 2008), and hIP1 (Choi et al., 2006) contributed to left inferior parietal activation. Superior parietal activation was present bilaterally in regions 7A and 7P (Scheperjans et al., 2008a,b). We also observed 3 frontal clusters in right dorsolateral prefrontal cortex (dlPFC), left dlPFC, and right inferior frontal cortex, including Brodmann Area 44 (Amunts et al., 1999).

**Table 1:** Anatomic locations of activation of quantifier conditions relative to precise number baseline.

Local maximum in macroanatomic structure	MNI coordinates			*Z* score	Cluster size (voxels)	Supporting cytoarchitectonic area(s)^*^
	x	y	z			
R inferior parietal	42	-44	48	5.14	1390	PFm, PGa
L superior parietal	-12	-68	58	4.04	713	7A, 7P
R middle frontal (dorsolateral prefrontal)	40	36	24	3.96	702	
R superior parietal	6	-66	58	3.8	899	7A, 7P
L inferior parietal	-58	-40	40	3.42	1264	PF, hIP1, PFm PGa
L middle frontal (dorsolateral prefrontal)	-44	52	8	3.37	548	
R inferior frontal	46	16	40	3.22	408	Area 44

* Cytoarchitectonic areas (Amunts et al., 1999; Choi et al., 2006; Caspers et al., 2008; Scheperjans et al., 2008a,b) were considered “supporting” if >10% of the activated cluster was in the region.

**FIGURE 3:**
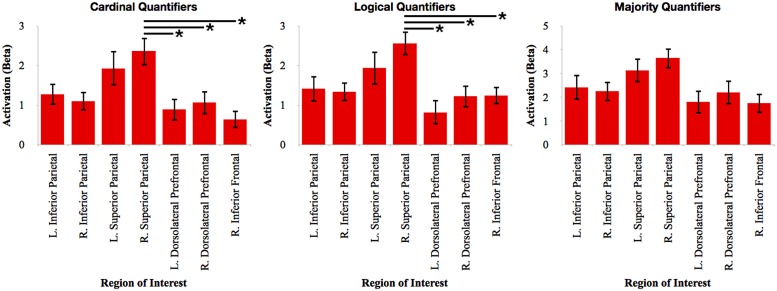
**Relative activation of each region of interest identified in the whole-brain analysis for each independent quantifier.** Bars with asterisks (*) represent *p* < 0.05 Bonferroni-corrected.

To evaluate the relative contribution of each of these clusters, we calculated the mean beta value in each ROI during the activation associated with each quantifier. We first performed an ANOVA with Bonferroni-corrected *post hoc t*-test contrasts for each quantifier class to evaluate the relative network activation associated with each quantifier (see **Figure 3**). For cardinal quantifiers [*F*(6,112) = 3.90, *p* = 0.001], we observed significantly increased activation in right superior parietal cortex relative to right inferior frontal (*p* = 0.003), left dlPFC (*p* = 0.022), and marginal right dlPFC (*p* = 0.078). For logical quantifiers [*F*(6,112) = 3.43; *p* = 0.004], we also observed increased right superior parietal activation relative to each of the frontal regions, including left dlPFC (*p* = 0.002), right dlPFC (*p* = 0.05), and marginal right inferior frontal (*p* = 0.065). While we observed an effect approaching significance for majority quantifiers [*F*(6,112) = 2.17, *p* = 0.051], *post hoc* contrasts did not reveal any significant differences across regions, suggesting that frontal regions were activated as robustly as parietal activations for majority quantifiers. Together, these findings suggest the relatively increased activation of superior parietal cortex for cardinal and logical quantifiers compared to frontal cortex and additionally highlight relatively equal amounts of frontal and parietal activation for majority quantifiers.

**FIGURE 4:**
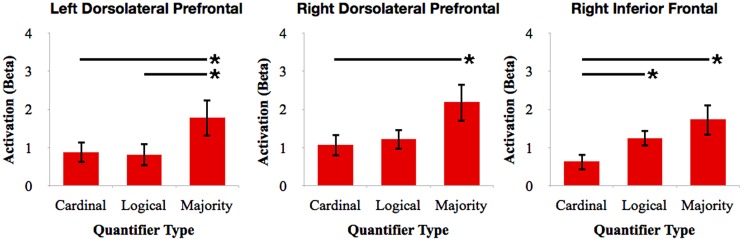
**Relative activation of ROIs in frontal cortex for cardinal, logical, and majority quantifiers.** Bars with asterisks (*) represent *p* < 0.05 Bonferroni-corrected.

To further investigate the relative involvement of frontal cortex for quantifier comprehension, we performed pairwise comparisons between types of quantifiers within each frontal ROI (see **Figure 4**). We performed paired-samples *t*-tests with Bonferroni correction. We observed increased right dlPFC activation for majority quantifiers relative to cardinal quantifiers [*t*(16) = 3.42; *p* = 0.027]. We also observed increased right dlPFC and left dlPFC activation for majority quantifiers relative to logical quantifiers [right: *t*(16) = 3.17; *p* = 0.05; left: *t*(16) = 3.19; *p* = 0.05]. We additionally observed increased right inferior frontal cortex activation for logical quantifiers relative to cardinal quantifiers [*t*(16) = 3.46; *p* = 0.027]. In summary, these comparisons across quantifier classes suggest that majority quantifier comprehension is supported by bilateral dlPFC in comparison to other quantifier classes, and that logical quantifier comprehension is additionally supported by right inferior frontal cortex.

Some of our clusters spanned across multiple cytoarchitectonic areas. For these clusters, we investigated whether there were differences in activation between constituent sub-regions. For each condition in each macroanatomical structure, we performed an ANOVA with Bonferroni-corrected* post hoc t*-test contrasts comparing the mean activation of the cytoarchitechonic areas and found no significant effects.

## DISCUSSION

There is increasing evidence that parietal and frontal cortices contribute to the comprehension of quantifiers (McMillan et al., 2005, 2006, 2013; Troiani et al., 2009a,b; Morgan et al., 2011; Heim et al., 2012). In this study, we identified a large-scale network in parietal and frontal cortex that supports the comprehension of multiple classes of quantifiers, and we evaluated the relative contribution of these components to each class of quantifier. Within the fronto-parietal network recruited by all quantifiers, we observed that dlPFC is up-regulated during the comprehension of majority quantifiers compared to other quantifier classes and that right inferior frontal cortex is recruited for the comprehension of logical quantifiers compared to cardinal quantifiers. We also found that right superior parietal cortex is more strongly activated than frontal cortical regions for logical and cardinal quantifiers. Inferior parietal cortex was activated equally often for all types of quantifiers.

The fronto-parietal processing system for quantifiers has been demonstrated in previous fMRI studies (McMillan et al., 2005; Troiani et al., 2009a). Even though we are studying the meaning of words, we argued that these findings cannot be easily attributed to activation of the core language processing system since the brain regions implicated in quantifier comprehension are not in left peri-Sylvian brain regions. Indeed, many of the activations are in the right hemisphere. We reasoned instead that quantifier comprehension depends in part on number knowledge because of the overlap of the inferior parietal regions thought to contribute to both quantifier comprehension and number knowledge. We used very small numbers in the present study to minimize the potential confound that larger numbers may depend in part on verbal mediation of precise number processing (Dehaene, 1997; Feigenson et al., 2004). Our baseline condition consisted of precise numbers describing the same pictures, a high-level baseline that further diminishes the possibility that the activations we are associating with quantifier processing are due to verbally mediated use of numbers or to visuo-spatial processing associated with picture stimuli. Finally, these activated frontal and parietal regions do not encompass brain regions typically thought to be engaged in core language processing. Additional evidence associating parietal regions with quantifier comprehension comes from patient studies, where quantifier comprehension deficits are found in non-aphasic patients who have parietal disease and whose performance is correlated with impaired number knowledge (McMillan et al., 2006; Troiani et al., 2009a; Morgan et al., 2011). These findings are broadly consistent with a model of semantic memory related to embodied cognition (Barsalou, 2008), where the meaning of a quantifier depends in part on the representation of quantity and magnitude information subserving an approximate number system in semantic memory.

Within this fronto-parietal network, we observed unique patterns of activation for each class of quantifier. This is consistent with the hypothesis that there are several partially distinct classes of quantifiers. In previous patient studies, we related cardinal quantifiers to gray matter atrophy in parietal cortex, while majority quantifiers were associated with atrophy additionally in dorsolateral prefrontal cortex (Troiani et al., 2009a; Morgan et al., 2011). Logical quantifiers were related to disease in more anterior portions of the frontal lobe in these studies. Below we discuss the unique combinations of these frontal and parietal regions in the neural representation of this fMRI study examining quantifier comprehension.

Parietal cortex is often implicated as playing a critical role for supporting number knowledge, broadly including the ability to perform number identification, counting, calculations, number magnitude comparisons, and other similar tasks, in fMRI (Dehaene et al., 2003; Piazza et al., 2007; Wei et al., 2012) and patient (Cipolotti et al., 1991; Takayama et al., 1994; Martory et al., 2003; Halpern et al., 2004; Baldo and Dronkers, 2007; Koss et al., 2010) studies. Previous fMRI studies of quantifier comprehension have also consistently reported parietal cortex activation (McMillan et al., 2005; Troiani et al., 2009a; Heim et al., 2012) and patients with neurodegenerative disease in parietal cortex have difficulty with interpreting quantifier meaning (McMillan et al., 2005, 2006; Morgan et al., 2011; Troiani et al., 2009a). Together, these findings have been interpreted as evidence for the role of number knowledge contributing to quantifier comprehension. Furthermore, consistent with conceptual processing theories (Binder et al., 1999), linguists have proposed similarities between number knowledge and quantifiers, regardless of the inclusion of an explicit number component (Barwise and Cooper, 1981; van Benthem, 1986). Neuroimaging studies have supported this notion and found inferior parietal cortex activation when processing quantifiers without explicit number components, such as logical quantifiers (McMillan et al., 2005; Wei et al., 2012). This suggests that though number knowledge itself is not required specifically for the processing of a logical quantifier, logical quantifiers are semantically related to cardinal quantifiers and majority quantifiers that require number knowledge. Thus, the processing of logical quantifiers may draw upon cognitive resources similar to those used when processing cardinal quantifiers and majority quantifiers.

The precise locus of parietal activation associated with quantifier comprehension has varied from study-to-study. One study reported that both inferior parietal and superior parietal cortices are activated during quantifier comprehension and that the relative magnitude of activation for each region was related to specific task demands (Heim et al., 2012). Thus, superior parietal cortex was recruited relative to the magnitude of target items, while inferior parietal was up-regulated in proportion to processing difficulty as measured by response latencies. In a different study it was suggested that inferior parietal is more related to number knowledge associated with cardinal quantifiers while superior parietal cortex including posterior cingulate is more related to the spatial distribution of attention during comprehension of logical quantifiers (Troiani et al., 2009a). However, the latter study did not control for response latencies and thus it is difficult to interpret whether inferior parietal cortex activation was confounded by longer response latencies for cardinal compared to logical quantifiers. In the current study, we discovered a significant difference between the response latencies of different quantifier classes. In hopes of examining differences in the neuroanatomical patterns of activation during the processing of each type of quantifier, as opposed to examining differences in difficulty of quantifier processing, we factored out response latencies as a nuisance covariate in our whole-brain analysis. We observed that inferior parietal cortex was equally activated across quantifier classes. Thus, inferior parietal cortex appears to contribute a core component of quantifier comprehension that cannot be easily explained by task-related attributes reflected in response latencies. Right superior parietal cortex was up-regulated in comparison to frontal cortex for cardinal and logical quantifiers. This may be related in part to the spatial distribution of attention when viewing a visual array for specific attributes to which we must attend when evaluating the truth-value of a quantifier (Corbetta et al., 1995; Corbetta and Shulman, 2002). Future research is required to assess the contribution of superior parietal cortex.

In addition to parietal cortex, prefrontal cortex has been implicated in the comprehension of specific classes of quantifiers. For example, majority quantifiers are hypothesized to require additional executive resources, such as working memory and strategic planning, in order to evaluate the quantity of target items in a set, maintain this numerosity in an active cognitive state while the quantity of total items in a set is evaluated, and then to compare these quantities. Non-aphasic patients with bvFTD have disease in dlPFC and have difficulty with majority quantifier comprehension despite relatively preserved cardinal quantifier comprehension (McMillan et al., 2006; Troiani et al., 2009a; Morgan et al., 2011). FMRI activation in dlPFC also was reported previously for majority quantifier comprehension in healthy adults (McMillan et al., 2005; Troiani et al., 2009a). Our observation in the current study of selective bilateral dlPFC activation for majority quantifier comprehension relative to cardinal and logical comprehension is consistent with these prior reports.

Right inferior frontal cortex was selectively activated for logical quantifiers compared to cardinal quantifiers. Right inferior frontal cortex was also previously implicated in quantifier processing (Heim et al., 2012). Inferior frontal cortex has been associated with many processes, including a role in selection within semantic memory (Thompson-Schill, 2003). However, other work has observed rostral prefrontal activation for logical quantifiers (Troiani et al., 2009a) and patients with disease in rostral prefrontal cortex have difficulty with logical quantifier interpretation (Morgan et al., 2011). The basis for this discrepancy is unclear. Other researchers have proposed that right inferior frontal cortex has a role in controlling attention (Corbetta and Shulman, 2002), as would be required to discriminate between the objects of interest and the background in our study. While previous work examining quantifier comprehension has used simple objects and colored circles independent of a contextualizing background, the current study probed quantifier comprehension in the context of complex scenes, possibly resulting in the inferior frontal activation we observed. Similarly, when processing cardinal quantifiers, focus may be drawn to a location of interest defined by the prompt; yet, when considering a logical quantifier that requires a single exception from the stated condition to confirm the truth-value of the condition, processing of the entire scene, as opposed to a particular region, is required. Additional work will be needed to investigate the basis for this difference.

Earlier work examining quantifiers has also shown activation in left inferior frontal cortex that we did not find in the current study (McMillan et al., 2005). In one instance, left inferior frontal cortex was activated when comparing “approximate” judgments to “precise” judgments (McMillan et al., 2005). Presently, however, a “precise quantifier” is defined to mean exclusively conditions in which the quantifier is a number, whereas previously “precise judgment” referred to any condition in which the target objects must be counted in order to determine the scene’s relation to the target sentence. Further, we did not present stimuli with numerosity discrepancies as large as those presented in the past work, potentially decreasing our sensitivity to the left inferior frontal activation associated with approximate judgments that were found previously.

In conclusion, this fMRI study adds to the mounting evidence suggesting that a large-scale fronto-parietal network contributes to quantifier comprehension. We additionally demonstrated that parietal cortex selectively supports cardinal and logical quantifier comprehension, dlPFC selectively supports majority quantifier comprehension, and right inferior frontal cortex supports logical quantifier comprehension. Together, these findings emphasize the relative importance of distinct neuroanatomical mechanisms for supporting distinct semantic classes of quantifiers.

## Conflict of Interest Statement

The authors declare that the research was conducted in the absence of any commercial or financial relationships that could be construed as a potential conflict of interest.
